# The Effect of Chinese Herbal Medicine on Quality of Life and Exercise Tolerance in Heart Failure With Preserved Ejection Fraction: A Systematic Review and Meta-Analysis of Randomized Controlled Trials

**DOI:** 10.3389/fphys.2018.01420

**Published:** 2018-10-26

**Authors:** Jinping Wang, Ran Yang, Feilong Zhang, Caixia Jia, Peipei Wang, Junjie Liu, Kuo Gao, Hua Xie, Juan Wang, Huihui Zhao, Jianxin Chen, Wei Wang

**Affiliations:** ^1^Dongzhimen Hospital, Beijing University of Chinese Medicine, Beijing, China; ^2^Guanganmen Hospital, China Academy of Chinese Medical Sciences, Beijing, China; ^3^Beijing University of Chinese Medicine, Beijing, China

**Keywords:** Chinese herbal medicine, heart failure, preserved ejection fraction, quality of life, exercise tolerance, systematic review, meta-analysis

## Abstract

**Background:** Chinese herbal medicine (CHM) has a good effect of alleviating symptoms and improving quality of life and exercise tolerance in patients with heart failure with preserved ejection fraction (HFpEF), but it wasn't sufficiently valued and promoted because of the lack of evidence-based medical evidence.

**Aim:** To systematically review the effect of CHM on quality of life and exercise tolerance in patients with HFpEF.

**Methods:** We conducted a systematic literature search for Chinese and English studies in PubMed, EMBASE, Cochrane Central Register of Controlled Trials, Chinese Biomedical Literature Database, China Knowledge Resource Integrated Database, Wanfang Data, and China Science and Technology Journal Database. Databases were searched using terms relating to or describing CHM, HFpEF and randomized controlled trials, without any exclusion criteria for other types of diseases or disorders. Literature retrieval, data extraction, and risk of bias assessments were performed independently by two investigators. Differences were resolved by consensus. RevMan 5.3.0 was used for data analysis. Quantitative synthesis was used when the included studies were sufficiently homogeneous and subgroup analyses were performed for studies with different sample sizes and blind methods. GRADEpro was used to grade the available evidence to minimize bias in our findings.

**Results:** Seventeen studies with 2,724 patients were enrolled in this review. ROB assessments showed a relatively high selection and performance bias. Meta-analyses showed that compared with conventional western medicine, combined CHM and conventional western medicine could significantly improve 6-min walk distance (MD = 52.13, 95% CI [46.91, 57.34], *P* < 0.00001), and it seemed to be more effective as compared with combined placebo and conventional western medicine. Similar results were observed for quality of life and the results were better in a larger sample. The GRADEpro showed a very low to moderate level of the available evidence.

**Conclusion:** Combined CHM and conventional western medicine might be effective to improve exercise tolerance and quality of life in HFpEF patients, but new well-designed studies with larger sample size, strict randomization, and clear description about detection and reporting processes are needed to further strengthen this evidence.

## Introduction

With a morbidity of 41 to 70% of all heart failure(van Riet et al., 2014, 2016), heart failure with preserved ejection fraction (HFpEF) has a similar quality of life and readmission rate with heart failure with reduced ejection fraction (HFrEF) (Farr et al., 2008; Ponikowski et al., 2016).

It has been shown that diuretics can alleviate the clinical symptoms of HFpEF (Faris et al., 2002, 2012), but the efficacy of beta blockers, ACEI, ARB, and mineralocorticoid receptor antagonists in HFpEF is inconclusive (Yusuf et al., 2003; Cleland et al., 2006; Massie et al., 2009). Worse still, no drugs have been conclusively shown to reduce the morbidity or mortality of the patients with HFpEF (Ponikowski et al., 2016; Yancy et al., 2017). Since these patients are often elderly and highly symptomatic with a poor quality of life (Fukuta et al., 2016), an important aim of therapy may be to alleviate symptoms and improve well-being. Traditional Chinese medicine has a unique effect on the treatment of heart failure (Li et al., 2013; Hao et al., 2017). A considerable number of randomized clinical trials (RCTs) have also shown that Chinese herbal medicine (CHM) has a good effect of alleviating symptoms and improving quality of life and exercise tolerance in patients with HFpEF. With lower cost and fewer side effects, CHM has been considered as an adjunctive treatment for chronic heart failure in some areas of China (Wang et al., 2017). However, in the world, especially in western countries, CHM has not been paid enough attention and widely used to treat HFpEF, due to the lack of evidence-based medical evidence. Therefore, it is necessary to conduct a meta-analysis of RCTs on the effect of CHM on quality of life and exercise tolerance in patients with HFpEF to provide evidence for the application of CHM for HfpEF worldwide.

## Methods

This meta-analysis was performed and reported according to the Preferred Reporting Items for Systematic Reviews and Meta-Analyses (PRISMA) (Moher et al., 2009). No published study protocol exists for this meta-analysis.

### Definition of heart failure with preserved ejection fraction

While there is clear agreement that the diagnosis of HFrEF requires an LVEF < 40%, the exact definition of HFpEF is less clear. According to the definition provided in the latest European Society of Cardiology guidelines, the diagnosis of HFpEF requires an LVEF ≥50%, whereas patients with LVEF between 40 and 49% are considered to have heart failure with mid-range ejection fraction (HFmrEF) (Ponikowski et al., 2016). The American College of Cardiology defined HFpEF as an LVEF >40%, with anything from 41 to 49% as borderline HFpEF (Yancy et al., 2013). Clinically, patients with HFmrEF have generally been included in trials of HFpEF, and RCTs have used various LVEF cut-offs, ranging from 40 to 50%. Accordingly, data summarized in this meta-analysis will include patients in the mid-range and borderline group. Therefore, in this study, LVEF ≥40% will be referred to as HFpEF.

### Inclusion criteria

1. Participants: There were no restrictions on patients' age, gender, disease duration, case source, nationality, or race. In the original literature, patients should have had a clear diagnosis of HFpEF. The sample size of each group should be ≥60. Besides, there were no restrictions with respect to other types of diseases or disorders.
2. Intervention: All types of CHM, either alone or in combination with other treatment for HFpEF, regardless of the dose, method of dosing, the composition of the formulae or duration of administration; were compared with other treatments. The following comparisons were studied: (1) CHM vs. placebo; (2) CHM vs. no treatment; (3) CHM alone vs. other pharmaceuticals (mainly Western medicines); (4) Combined CHM and other pharmaceuticals vs. other pharmaceuticals (mainly Western medicines); (5) Combined CHM and other pharmaceuticals vs. combined placebo and other pharmaceuticals (mainly Western medicines).
3. Control: As talked above, the control could be placebo, no treatment, other pharmaceuticals, or combined placebo and other pharmaceuticals.
4. Outcomes: Primary outcomes were quality of life as measured using the Minnesota Living With Heart Failure Questionnaire (MLHFQ) (Rector and Cohn, 1992) or the Medical Outcomes Study Short Form 36-Item Health Survey (SF-36) (Ware and Gandek, 1998), and exercise tolerance as measured using 6-min walk distance (6MWD). Secondary outcomes were all-cause mortality, Heart failure hospitalization, clinical efficacy rate as measured using Lee's Criteria for Determining Heart-Failure Score(Lee et al., 1982) or cardiac function class of NYHA, as defined in the Guidelines for clinical research of new Chinese medicine drugs (Zheng, 2002), and biomarkers [B-type natriuretic peptide (BNP), N-terminal pro-B-type natriuretic peptide (NT-proBNP)]. Clinical efficacy rate as measured using Lee's Criteria for Determining Heart-Failure Score included the following: (1) Significant effect: Lee's scores decreased more than 75% after treatment; (2) Effective: points of Lee's Criteria for Determining Heart-Failure Score reduced by 50 to 75% after treatment; (3) Invalid: after treatment, the score decreased by < 50%; (4) Aggravation: the score after treatment exceeded the score before treatment. Clinical efficacy rate as measured using cardiac function class of NYHA were defined as follows: (1) Significant: heart failure was basically controlled or cardiac function was increased by 2 levels or above; (2) Effective: cardiac function increased by 1 level; (3) Invalid: cardiac function increased by < 1 level; (4) Impaired: cardiac function deteriorated by 1 level or more after treatment.
5. Study type: Only randomized controlled trials (RCTs) with parallel group design were included, whether published in full or abstract.

### Literature searches

A systematic search for Chinese and English studies was performed in the following databases: PubMed, EMBASE, Cochrane Central Register of Controlled Trials (CENTRAL), Chinese Biomedical Literature Database (CBM), China Knowledge Resource Integrated Database (CNKI), Wanfang Data, and China Science and Technology Journal Database (VIP). These databases were searched from the earliest date until April 2018, with terms relating to or describing CHM, HFpEF and randomized controlled trials, without any exclusion criteria for other types of diseases or disorders. An illustrative PubMed search strategy is shown below:

#1 (((((((“Medicine, Chinese Traditional”[Mesh]) OR “Medicine, East Asian Traditional”[Mesh]) OR “Herbal Medicine”[Mesh]) OR “Drugs, Chinese Herbal”[Mesh]) OR “Plants, Medicinal”[Mesh]) OR “Phytotherapy”[Mesh]) OR “Medicine, Kampo”[Mesh]) OR “Medicine, Korean Traditional”[Mesh]
#2 (((((“Heart Failure, Diastolic”[Mesh]) OR Diastolic Heart Failures) OR Heart Failures, Diastolic) OR Diastolic Heart Failure)) OR ((((((((((preserved) OR normal) OR greater)) AND (((ejection fraction) OR EF) OR LVEF))) OR ventricular dysfunction) OR ventricular function)) AND ((“Heart Failure”[Mesh]) OR (((((heart) OR myocardial) OR cardia^*^)) AND (((failure) OR insufficient^*^) OR decompensat^*^))))
#3 (((((((randomized controlled trial) OR controlled clinical trial) OR randomized) OR placebo) OR drug therapy) OR randomly) OR trial) OR groups
#4 (“Animals”[Mesh]) NOT “Humans”[Mesh]
#5 #1 AND #2 AND #3 AND #4

**Figure 1:**
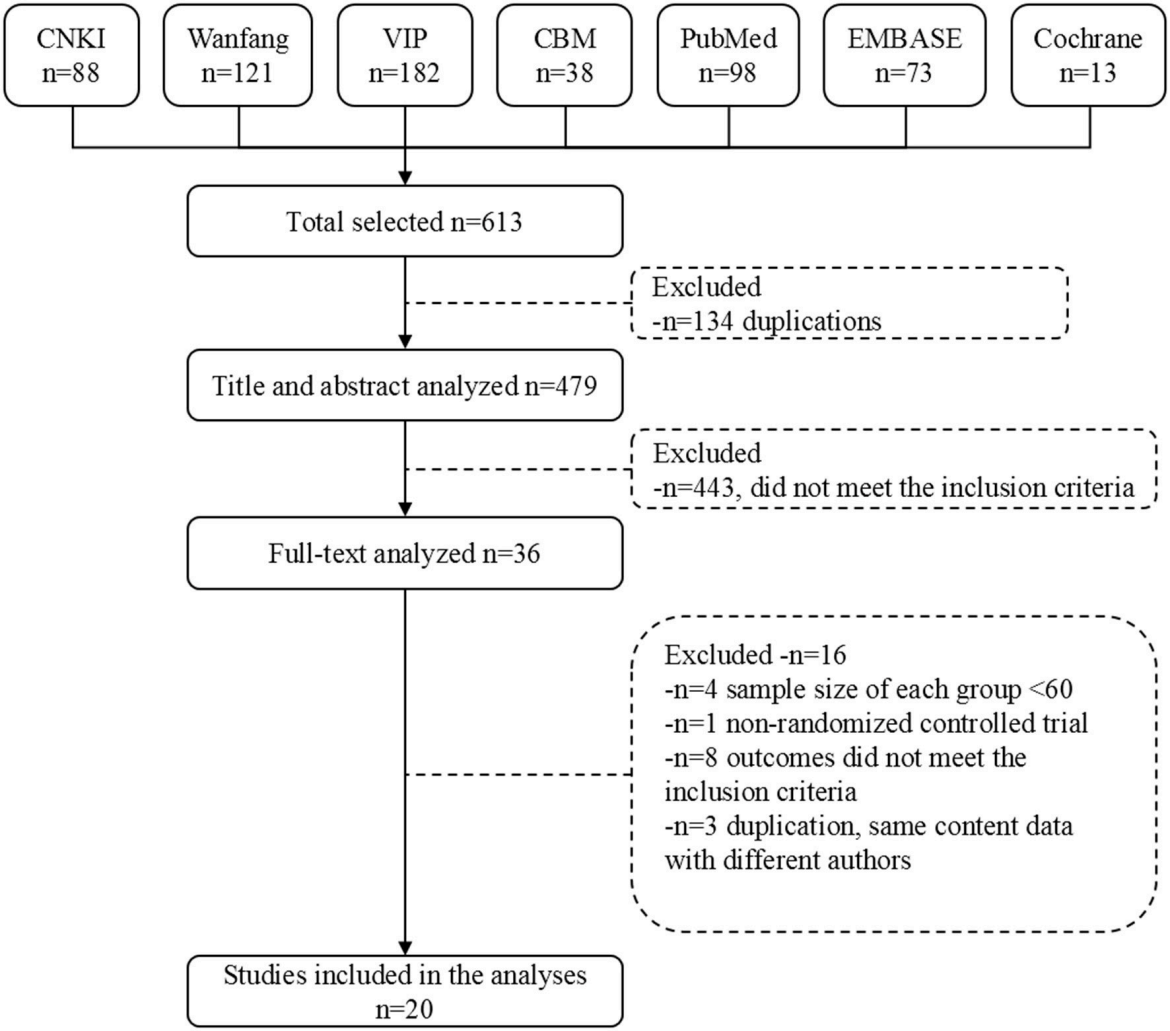
Literature screening process.

After removal of duplicates, the title and abstracts of initial search results were independently screened for relevance by two investigators. Abstracts that did not meet the eligibility criteria were excluded, and those that did not provide sufficient information about the inclusion criteria were further reviewed. The full texts of remaining results were further assessed by the same investigators, blinded to each other's review. All differences were resolved by consensus. Study selection flow diagram is shown in Figure 1.

### Data extraction

Data were extracted independently and in duplicate by two investigators, and were transcribed onto a dedicated database. The data extracted from each report included basic information (study ID, document type, author, publishing year); participants' demographic details (sample size, age, and gender), study diagnostic criteria, inclusion criteria, exclusion criteria; study drug and control treatment; outcomes, fall outs; follow-up duration and outcome events. Disagreements in abstracted data were adjudicated by a third reviewer (AAN).

### Risk of bias assessment

The Cochrane Collaboration risk of bias tool was used to assess risk of bias.

The methodological quality of the included studies was assessed by two investigators independently using RevMan 5.3.0, in the light of Cochrane Handbook criteria for judging ROB in the “Risk of bias” assessment tool. Judgment on the risk of bias was graded as low, unclear, or high ROB in the following aspects: random sequence generation (selection bias), allocation concealment (selection bias), blinding of participants and personnel (performance bias), blinding of outcomes assessment (detection bias), incomplete outcome data (attrition bias), selective reporting (reporting bias), and other bias. Again, all differences were resolved by consensus.

### Statistical analyses

RevMan 5.3.0 provided by the Cochrane Collaboration was used to analyze the results of the studies. For binary outcomes, estimates were summarized as relative risk (RR) and 95% CI. For continuous outcomes, weighted mean difference (MD) or standard mean difference (SMD) and 95% CIs were calculated. The complete case data were used as the analysis data. Heterogeneity between the studies in effect measures was assessed using both the Chi-squared test and the I-squared statistic (Higgins et al., 2003) with an I-squared value >50% indicative of substantial heterogeneity. Quantitative synthesis was used when the included studies were sufficiently homogeneous, both statistically and clinically. When the I-squared value was lower than 30% and *P* > 0.10, a fixed-effect model was used; otherwise, a random effects model was used.

Separate subgroup analyses for studies with different sample sizes and blind methods were performed because of the significant heterogeneity of primary outcomes. As the methodological quality of the included studies was generally low, and the results of the subgroup analyses showed significant positive results, no further sensitivity analyses were carried out.

Since the number of studies in each project is <10, no funnel plot was generated to detect publication bias. GRADEpro online summary of findings table for outcomes was used to grade the available evidence to minimize bias in our findings and recommendations. The judgement included bias risk, inconsistency (heterogeneity), indirect, imprecision, and publication bias; the level of each evidence was graded as very low, low, moderate, or high.

## Results

### Literature screening

We retrieved 613 original literatures from electronic bibliographic databases published from 1982 to 2017. After 134 duplications being excluded, 479 publications were screened and evaluated for eligibility based on title and abstract only, whereby another 443 papers that did not meet the inclusion criteria were excluded. We downloaded the full text of the remaining 36 publications for future screening. Finally, 20 articles were included for meta-analysis, comprising data from 17 trials (Figure 1).

### Characteristics of included studies

The 20 articles were published between 2009 and 2017, including 14 trials of combined oral Chinese herbal medicines (including decoctions, granules and capsules) and western medicines, and 3 trials of combined traditional Chinese medicine injections and western medicines. Controls included western medicines and combined placebo and western medicines. In total, 2,724 patients were randomized to either CHM intervention or control group, all of which were from China. The mean age of participants ranged from 53.6 to 76.5 years, and the proportion of men ranged from 44.2 to 63.3%. All trials were presented as full journal articles. Outcomes included all-cause mortality, Heart failure hospitalization, 6MWD, quality of life, clinical effective rate, BNP, NT-proBNP, and adverse events. The treatment duration lasted from 10 days to 3 months (Table 1).

**Table 1:** Characteristics of included studies.

**Studies**	**Total (N)**	**Entry-EF cut-off**	**Intervention group**			**Control group**			**Treatment duration**	**Outcomes**
			**Sample size**	**Age**	**Intervention**	**Sample size**	**Age**	**Control**		
Tian, 2011	120	50%	60	60.30 ± 7.36	Qili Qiangxin capsules, Benazepril hydrochloride tablets, Metoprolol tartrate tablets	60	61.02 ± 7.89	Benazepril hydrochloride tablets, Metoprolol tartrate tablets	1 month	BNP, clinical efficacy rate, MLHFQ, clinical efficacy rate
Chang and Gong, 2014	160	45%	80	62.41	Conventional western medicine, decoction of CHM (radix pseudostellariae, ligusticum wallichii, lilium brownii, dried rehmannia root, astragalus, root of common peony, angelica sinensis, radix ophiopogonis, the root of red-rooted salvia, herba leonuri)	80	61.32	Conventional western medicine	1 month	All-cause mortality, heart failure hospitalization
Zhou et al., 2014	160	45%	80	60.54	Conventional western medicine, decoction of CHM (radix pseudostellariae, dried rehmannia root, radix ophiopogonis, the root of red-rooted salvia, lilium brownii, astragalus, angelica sinensis, herba leonuri, ligusticum wallichii, root of common peony)	80	61.32	Conventional western medicine	1 month	BNP
Sun and Gong, 2013	160	45%	82	61.32 ± 5.4	Imidapril, Bisoprolol, Aldactone, Isosorbide dinitrate, Yi Qi Yang Yin Huo Xue decoction (scrophularia ningpoensis, radix ophiopogonis, dried rehmannia root, astragalus, angelica sinensis, ligusticum wallichii, root of common peony, the root of red-rooted salvia, radix codonopsis)	78	60.54 ± 5.08	Imidapril, Bisoprolol, Aldactone, Isosorbide dinitrate	30 days	Clinical efficacy rate
Liu et al., 2011a	120	50%	60	52.88 ± 0.34	Conventional western medicine, Bawei Tongluo granules (leech, lumbricus, ligusticum wallichii, the root of red-rooted salvia, the root of kudzu vine, peach seed, safflower, honey-fried licorice root)	60	54.32 ± 9.76	Conventional western medicine, Bawei Tongluo granulation simulator	8 week	Clinical efficacy rate, 6MWD, MLHFQ
Wang and Ouyang, 2016	210	50%	105	65.6 ± 7.9	Enalapril, Tongmai Baoxin Decoction (astragalus, the root of red-rooted salvia, herba leonuri, poria cocos, radix ginseng rubra, safflower, semen lepidii, sappanwood, cassia twig, atractylodes macrocephala koidz, radix ophiopogonis)	105	64.4 ± 8.1	Enalapril	3 month	6MWD, BNP, MLHFQ
Zhou and Hong, 2016	184	50%	94	64.34 ± 3.32	Conventional western medicine, Huoxue Lishui Decoction (astragalus, rhodiola rosea, root of common peony, ligusticum wallichii, the root of red-rooted salvia, semen lepidii, rhizoma alismatis, grifola)	90	63.15 ± 12.37	Conventional western medicine	1 month	6MWD, NT-pro BNP, clinical efficacy rate
Wei, 2016	120	45%	60	61.2 ± 8.1	Conventional western medicine, Qili Qiangxin Capsules, Trimetazidine Dihydrochloride Tablets	60	61.9 ± 7.5	Conventional western medicine	4 week	Clinical efficacy rate
Yang and Suo, 2015	120	45%	60	59.21 ± 4.75	Enalapril, Metoprolol, Qili Qiangxin Capsules	60	59.49 ± 4.82	Enalapril, Metoprolol	1 month	BNP
Wen, 2013; Yu, 2013	120	50%	60	61.37 ± 6.32	Conventional western medicine, decoction of CHM (astragalus, the root of red-rooted salvia, angelica sinensis, herba leonuri, ligusticum wallichii, root of common peony)	60	62.02 ± 6.35	Conventional western medicine	4 week	6MWD, BNP, all-cause mortality, heart failure hospitalization, clinical efficacy rate
Liu et al., 2016, 2017; Xie et al., 2016	120	45%	60	76.7 ± 7.7	Conventional western medicine, Jiawei Zhenwu decoction (radix aconiti carmichaeli, ginger, poria cocos, atractylodes macrocephala koidz, radix paeoniae alba, cassia twig, semen lepidii, rhodiola rosea, radix pseudostellariae, astragalus)	60	76.3 ± 7.0	Conventional western medicine	4 week	6MWD _‵_ BNP _‵_ SF-36 _‵_ MLHFQ
Zhang, 2015a	180	50%	90	60–85	Conventional western medicine, Wenxin Keli	90	60-87	Conventional western medicine	12 week	Clinical efficacy rate
Ji, 2017	130	45%	65	72.98 ± 2.69	Conventional western medicine, Jiawei Zhenwu decoction (radix paeoniae alba, radix rehmanniae preparata, poria cocos, herba aristolochiae, ginger, atractylodes macrocephala koidz, angelica sinensis, radix codonopsis)	65	73.34 ± 2.78	Conventional western medicine	21 days	SF-36, 6MWD
Shang, 2016	188	50%	94	72.2	Conventional western medicine, salvianolate, Shenmai injection	94	72.2	Conventional western medicine	2 month	Clinical efficacy rate
Han et al., 2016	150	45%	75	66.0 ± 4	Conventional western medicine, benazepril, Danshen Chuanxiongqin injection	75	68.0 ± 5	Conventional western medicine	14 days	NT-proBNP, clinical efficacy rate
Zhang, 2015b	300	45%	150	71.8 ± 4	Conventional western medicine, Shuxuetong injection	150	72.2 ± 3	Conventional western medicine	10 days	BNP, clinical efficacy rate
Liu et al., 2011b	182	50%	101	70.64 ± 9.29	Conventional western medicine, Yixinshu capsules	100	69.29 ± 7.01	Conventional western medicine	8 week	BNP

### Risk of bias assessment

**Figure 2:**
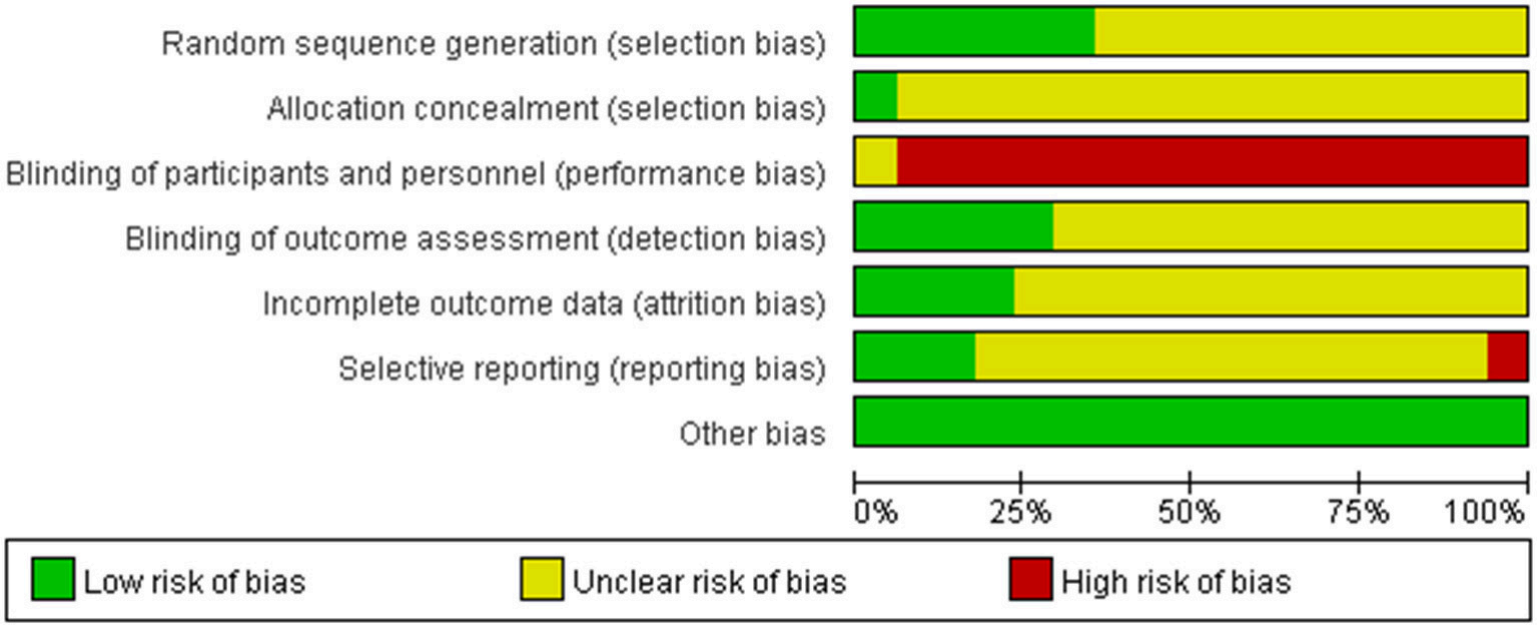
Risk of bias graph.

**Figure 3:**
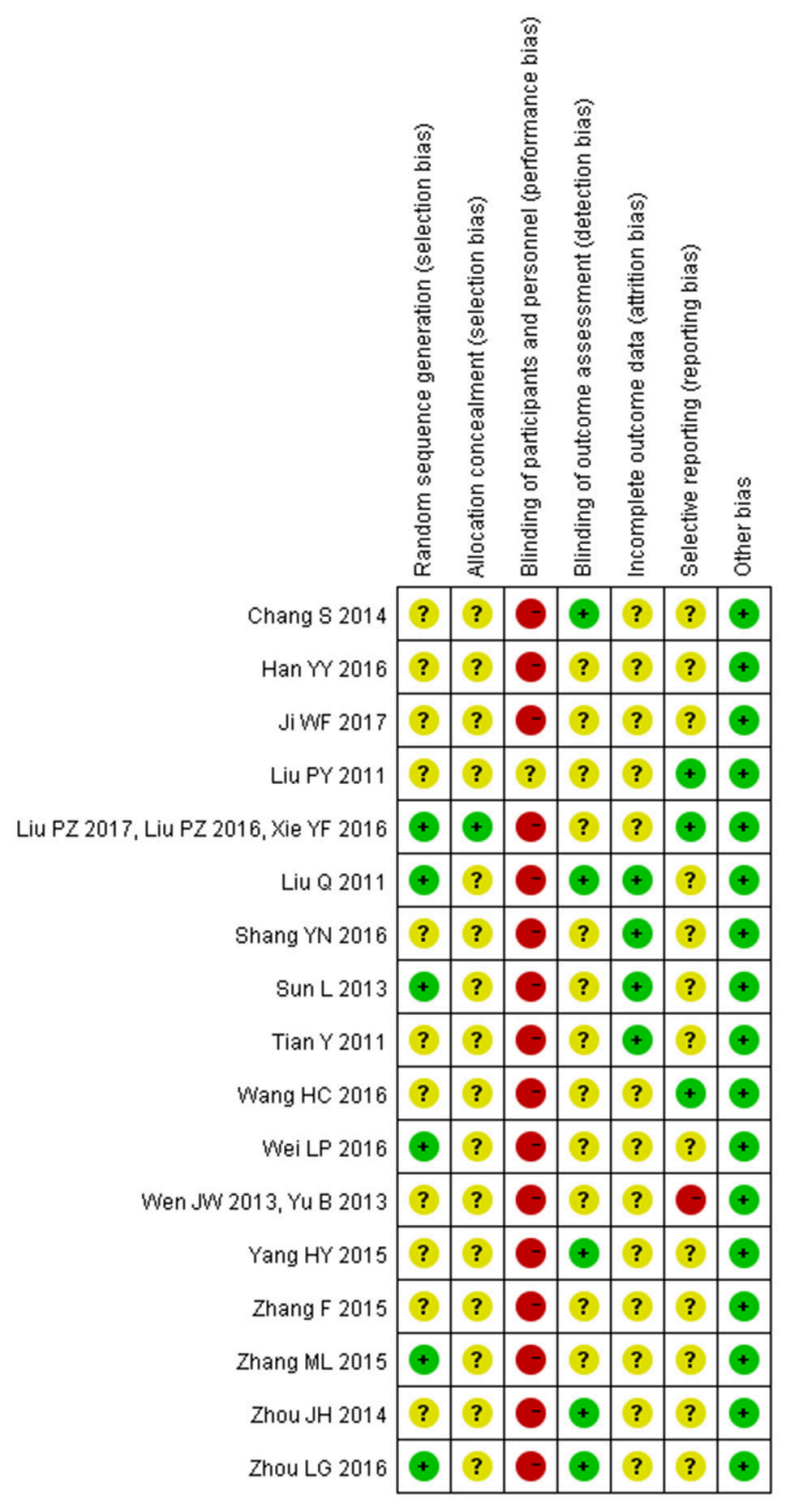
Risk of bias summary: review authors' judgements about each risk of bias item for each included study.

Two independent reviewers extracted the data of included studies and conducted a risk of bias assessment using the Cochrane Collaboration's tool for assessing risk of bias. In this review, all 17 trials were reported as randomized controlled trials. Of the 17, only seven reported the methods used for the generation of the allocation sequence. Among them, five used a random numbers table (Liu et al., 2011b, 2016, 2017; Tian, 2011; Sun and Gong, 2013; Wei, 2016; Xie et al., 2016) and two used random drawings (Zhang, 2015a; Zhou and Hong, 2016). Only one trial(Liu et al., 2016, 2017; Xie et al., 2016) provided information about allocation concealment. Only one trial reported use of single-blinding (Liu et al., 2011a). Three trials reported withdrawals(Liu et al., 2011b; Tian, 2011; Sun and Gong, 2013). No protocols of the included studies were available to us to investigate selective reporting. We assessed more than 3 studies(Liu et al., 2011a, 2016, 2017; Wang and Ouyang, 2016; Xie et al., 2016) included in this review to be at low risk for selective reporting, with the remaining 14 studies assessed as being at high risk. No other potential sources of bias could be found. The risk of bias in the included studies is shown in detail in Figures 2, 3.

## Efficacy analyses

### Effect of CHM on 6MWD

**Figure 4:**
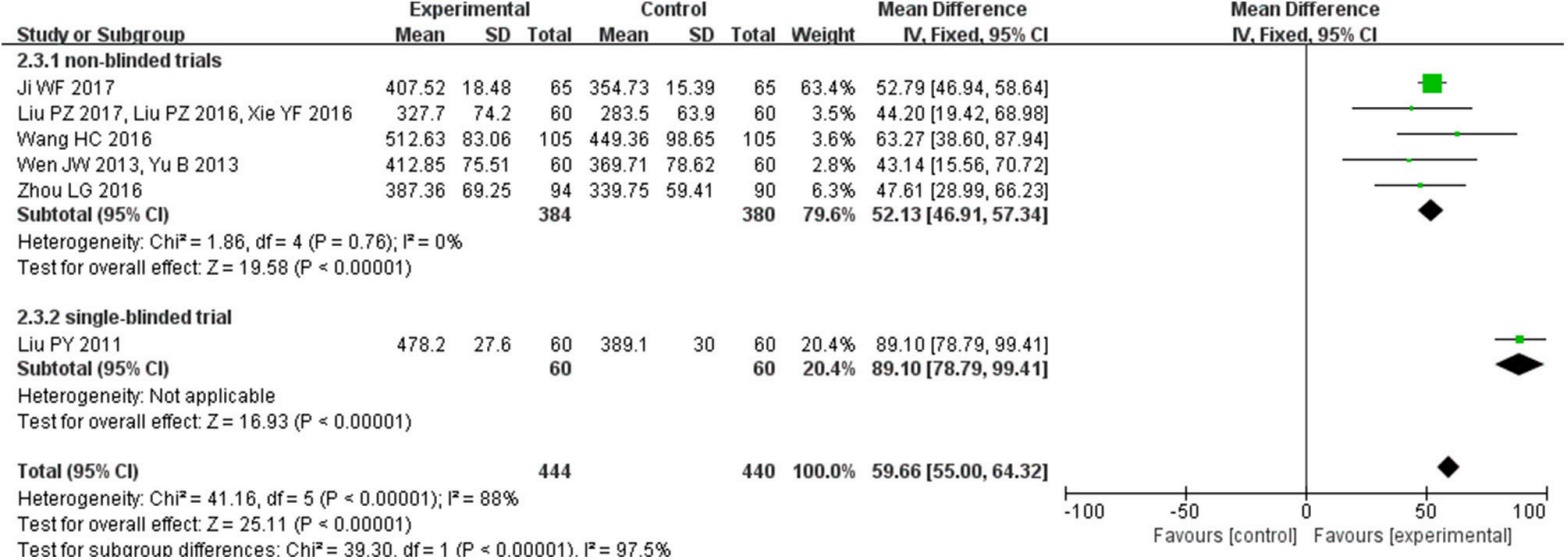
Pooled and individual estimates of mean difference (MD), and 95% CI of 6MWD for CHM and control therapies.

Six studies (*n* = 884 patients) reported on 6MWD. Meta-analysis showed that there was significant heterogeneity among the studies (*I*^2^ = 88%, *P* < 0.00001). Then, we found that one study (Liu et al., 2011a), the only single-blinded study, was a major source of heterogeneity. Therefore, we further conducted subgroup analyses according to different study types. For the five no-blinded studies, the in-group heterogeneity was small (*I*^2^ = 0%, *P* = 0.76), so we chose a fixed-effect model to do quantitative synthesis. Compared with conventional western medicine, CHM significantly improved 6MWD in HFpEF patients (MD = 52.13, 95% CI [46.91, 57.34], *P* < 0.00001). As to the single-blinded study, use of combined CHM and conventional western medicine also tended to be more effective in improving 6MWD than combined placebo and conventional western medicine (MD = 89.10, 95% CI [78.79, 99.41], *P* < 0.00001; Figure 4).

### Effect of CHM on quality of life

**Figure 5:**
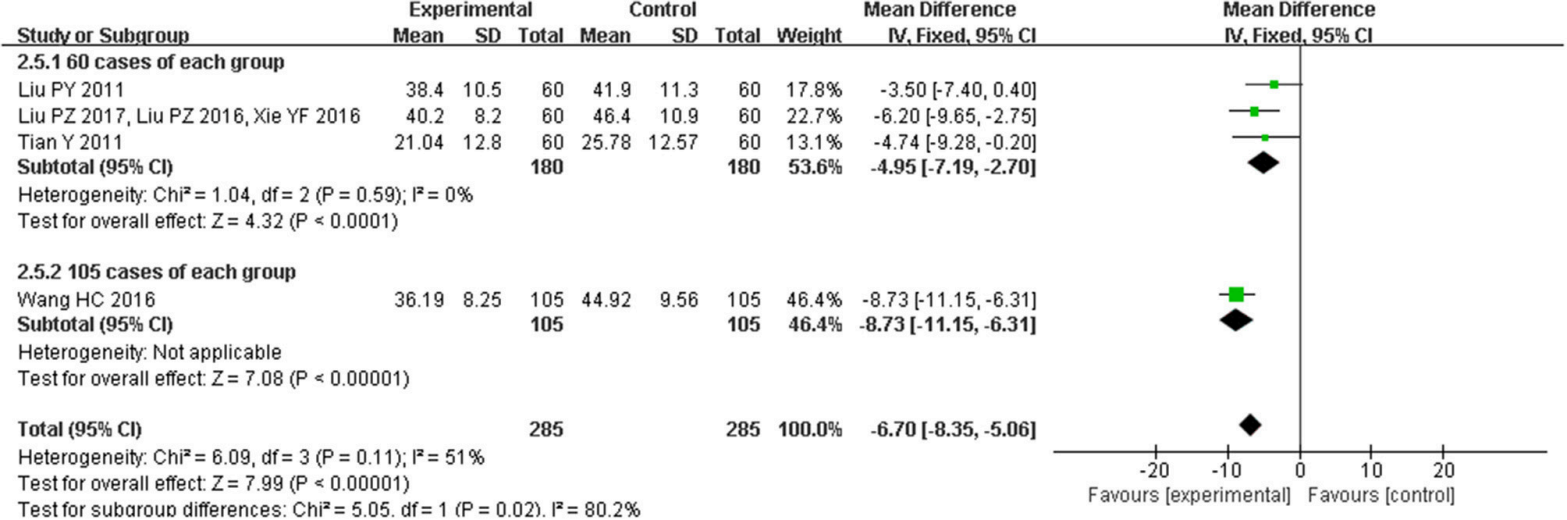
Forest plot of the comparison between CHM and control for quality of life as measured by the MLHFQ.

Four trials(Liu et al., 2011a, 2016, 2017; Tian, 2011; Wang and Ouyang, 2016; Xie et al., 2016) reported the treatment effects on quality of life as measured by the MLHFQ, including a total of 570 patients. There was substantial heterogeneity among the included studies (*I*^2^ = 51%, *P* < 0.00001), and we found that a trial of 210 patients (much larger than the sample size of other studies) was the primary source of heterogeneity. So, we conducted subgroup analysis based on sample size. Quality of life was improved in studies with the sample size of 120 (MD = −4.95, 95% CI [−7.19, −2.70], *P* < 0.0001), and the efficacy was better in the study with 210 participants (MD = −8.73, 95% CI [−11.15, −6.31], *P* < 0.00001; Figure 5).

**Figure 6:**
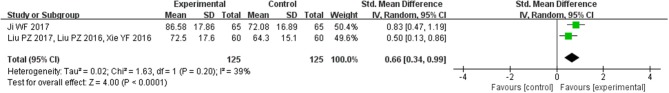
Forest plot of the comparison between CHM and control for quality of life as measured by the SF-36.

Two trials (Liu et al., 2016, 2017; Xie et al., 2016; Ji, 2017) with 150 patients talked about the treatment effects on quality of life as measured by the SF-36. Overall estimate showed that CHM resulted in better quality of life scores (SMD = 0.66, 95% CI [0.34, 0.99], *P* < 0.00001; Figure 6).

#### Effect of CHM on all-cause mortality and heart failure hospitalization

**Figure 7:**
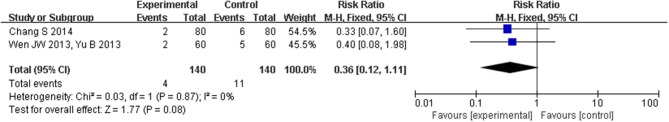
Forest plot of the comparison between CHM and control for all-cause mortality in 6 months after discharge.

The effect of CHM on mortality in HFpEF is shown in Figure 7. Only two studies (Wen, 2013; Yu, 2013; Chang and Gong, 2014) with 140 participants reported the effect of CHM on all-cause mortality in 6 months after discharge. The heterogeneity was small (*I*^2^ = 0%, *P* = 1.00), so we chose a fixed-effect model to do quantitative synthesis. Use of CHM was associated with reduced all-cause mortality in the pooled analysis, but this difference was not statistically significant (RR [95% CI] = −0.05 [−0.10, 0.00], *P*_fixed_ = 0.08).

**Figure 8:**
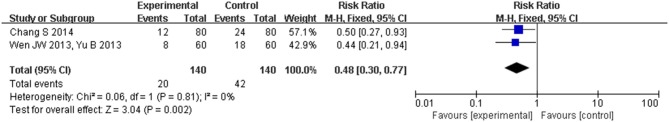
Forest plot of the comparison between CHM and control for heart failure hospitalization in 6 months after discharge.

The two studies (Wen, 2013; Yu, 2013; Chang and Gong, 2014) also reported the effects of CHM on heart failure hospitalization in 6 months after discharge. Compared with conventional western medicine for heart failure, CHM significantly reduced heart failure hospitalization (RR [95% CI] = −0.16 [−0.25, −0.06], *P*_fixed_ = 0.002; Figure 8).

### Effect of CHM on heart failure biomarkers

**Figure 9:**
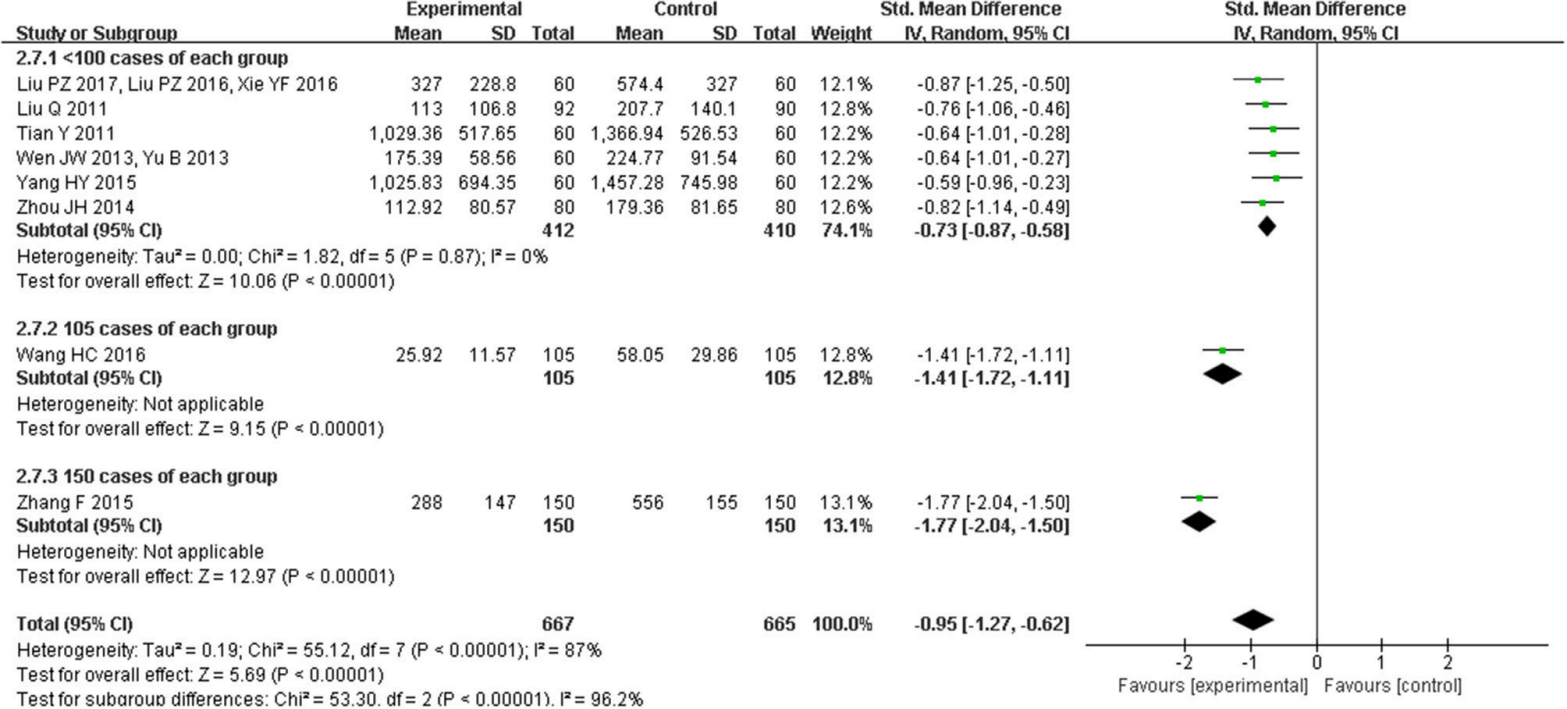
Pooled and individual estimates of standard mean difference (SMD), and 95% CI of BNP for CHM, and control therapies.

**Figure 10:**
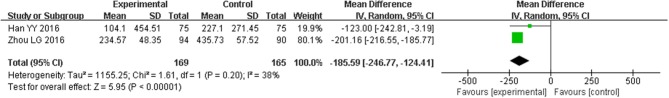
Pooled and individual estimates of mean difference (MD), and 95% CI of NT-proBNP for CHM, and control therapies.

Eight trials with 1332 participants reported BNP levels after treatment, and two trials of 434 patients mentioned NT-proBNP. The eight studies of BNP had a high degree of heterogeneity (*I*^2^ = 87%, *P* < 0.00001) and subgroup analyses on different sample sizes were further analyzed. BNP levels were significantly reduced in studies (Liu et al., 2011b, 2016, 2017; Tian, 2011; Wen, 2013; Yu, 2013; Zhou et al., 2014; Yang and Suo, 2015; Xie et al., 2016) with the sample sizes of < 200 participants (SMD = −0.73, 95% CI [−0.87, −0.58], *P* < 0.00001), which were more significant in the study (Wang and Ouyang, 2016) with 210 participants (SMD = −1.41, 95% CI [−1.72, −1.11], *P* < 0.00001) and the study (Zhang, 2015b) with 300 participants (SMD = −1.77, 95% CI [−2.04, −1.50], *P* < 0.00001; Figure 9). NT-proBNP levels were also significantly reduced (MD = −185.59, 95% CI [−246.77, −124.41], *P* < 0.00001) (Han et al., 2016; Zhou and Hong, 2016; Figure 10).

### Effect of CHM on clinical efficacy rate

**Figure 11:**
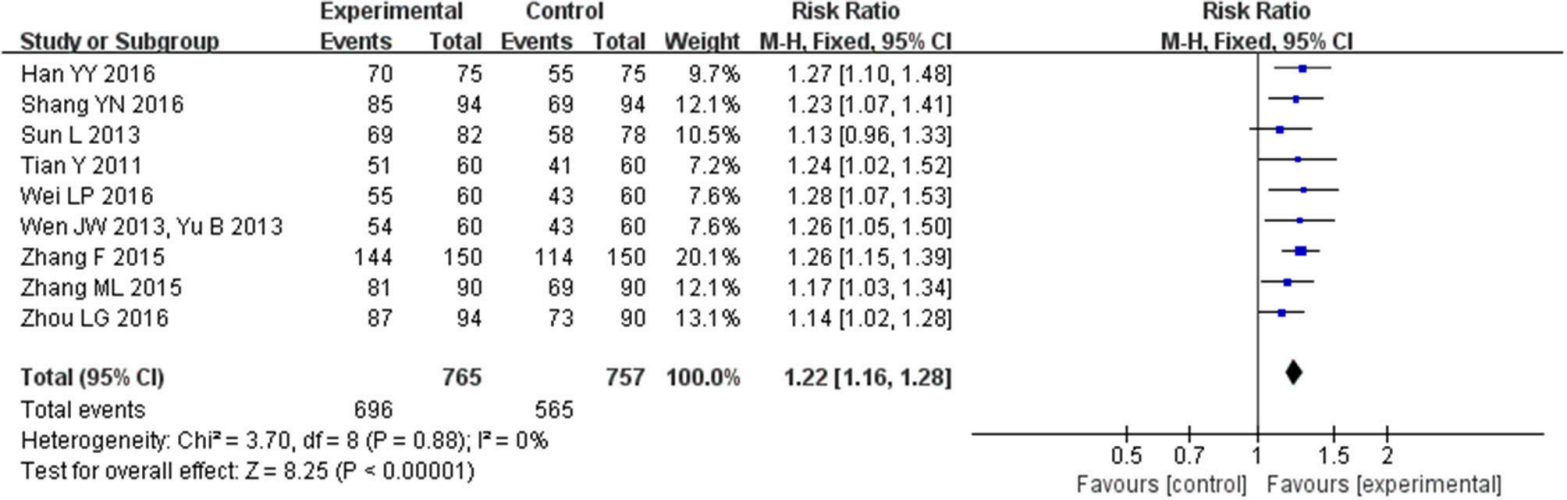
Forest plot of the comparison between CHM and control for clinical efficacy rate as measured using cardiac function class of NYHA as defined in the Guidelines for clinical research of new Chinese medicine drugs.

Nine trials (Tian, 2011; Sun and Gong, 2013; Wen, 2013; Yu, 2013; Zhang, 2015a,b; Han et al., 2016; Shang, 2016; Wei, 2016; Zhou and Hong, 2016) with 1522 participants mentioned clinical efficacy rate as measured using cardiac function class of NYHA as defined in the Guidelines for clinical research of new Chinese medicine drugs. The heterogeneity among the included studies was relatively small (*I*^2^ = 0%, *P* = 0.88); therefore, we carried out quantitative analysis using a fixed-effect model, which showed that CHM could greatly improve clinical efficacy rate of HFpEF (MD = 1.22, 95% CI [1.16, 1.28], *P* < 0.00001; Figure 11).

**Figure 12:**
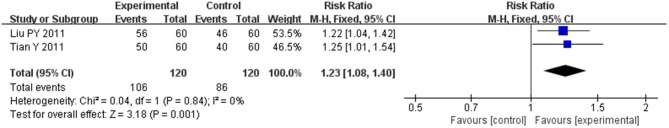
Forest plot of the comparison between CHM and control for clinical efficacy rate as measured using Lee's Criteria for Determining Heart-Failure Score.

Two trials (Liu et al., 2011a; Tian, 2011) of 240 patients reported clinical efficacy rate as measured using Lee's Criteria for Determining Heart-Failure Score. The heterogeneity between the two studies was also very small (*I*^2^ = 0%, *P* = 0.84), and we conducted quantitative analysis using a fixed-effect model as well, which also showed that CHM had a good effect in improving clinical efficacy rate of HFpEF (MD = 1.23, 95% CI [1.08, 1.40], *P* = 0.001; Figure 12).

### Adverse event

Of all the studies included, three studies(Liu et al., 2011b; Tian, 2011; Sun and Gong, 2013) mentioned losses to follow, and seven studies(Tian, 2011; Sun and Gong, 2013; Zhang, 2015a,b; Shang, 2016; Wang and Ouyang, 2016; Ji, 2017) reported adverse events. The initial recruitment had 2743 patients with HFpEF. There were only 19 cases lost to follow-up, none of which were due to a major cardiac event, and we collected complete data of 2724 cases (99.31%). Of the seven studies that reported adverse events, six reported no adverse events during the study, and one reported 2 cases of rashes in the CHM group, which healed quickly after treatment.

### GRADE evidence profile

Details of GRADE evidence profile and summary of finding table were given in Table 2. Because of serious risk of bias in study methods, heterogeneity and reporting bias, overall qualities of evidence for 6MWD, total scores of MLHFQ and SF-36 were judged as very low quality, low quality and moderate quality evidence, indicating that these estimates were uncertain and further studies are likely to have an impact on our confidence in the estimate of CHM effect.

**Table 2:** GRADEpro evidence grading.

**Certainty assessment**							**No of patients**		**Effect**		**Certainty**	**Importance**
**No of studies**	**Study design**	**Risk of bias**	**Inconsistency**	**Indirectness**	**Imprecision**	**Other considerations**	**CHM**	**control**	**Relative (95% CI)**	**Absolute (95% CI)**		
**6MWD**												
6	randomized trials	serious^a^	serious^b^	not serious	not serious	publication bias strongly suspected^c^	444	440	–	MD **59.66 higher** (55 higher to 64.32 higher)	⊕◯◯◯ VERY LOW	CRITICAL
**6MWD-NON-BLINDED TRIALS**												
5	randomized trials	serious^a^	not serious	not serious	not serious	publication bias strongly suspected^c^	384	380	–	MD **52.13 higher** (46.91 higher to 57.34 higher)	⊕⊕◯◯ LOW	CRITICAL
**6MWD-SINGLE-BLINDED TRIAL**												
1	randomized trials	not serious	not serious	not serious	not serious	none	60	60	–	MD **89.1 higher** (78.79 higher to 99.41 higher)	⊕⊕⊕⊕ HIGH	CRITICAL
**MLHFQ**												
4	randomized trials	serious^a^	serious^b^	not serious	not serious	none	285	285	–	MD **6.7 lower** (8.35 lower to 5.06 lower)	⊕⊕◯◯ LOW	CRITICAL
**MLHFQ-60 CASES OF EACH GROUP**												
3	randomized trials	serious^a^	not serious	not serious	not serious	none	180	180	–	MD **4.95 lower** (7.19 lower to 2.7 lower)	⊕⊕⊕◯ MODERATE	CRITICAL
**MLHFQ-105 CASES OF EACH GROUP**												
1	randomized trials	serious^a^	not serious	not serious	not serious	none	105	105	–	MD **8.73 lower** (11.15 lower to 6.31 lower)	⊕⊕⊕◯ MODERATE	CRITICAL
**SF-36**												
2	randomized trials	serious^a^	not serious	not serious	not serious	none	125	125	–	SMD **0.66 higher** (0.34 higher to 0.99 higher)	⊕⊕⊕◯ MODERATE	CRITICAL

CI, confidence interval; MD, mean difference; SMD, standardized mean difference.

a no-blinded.

b I^2^ > 50%.

c *selective reporting*.

### Biochemical/biological analysis on CHM and HFpEF

We also conducted a metabolic pathway analysis of the top 10 used CHMs in this study (the root of red-rooted salvia, astragalus, ligusticum wallichii, ginseng, semen lepidii, safflower, radix ophiopogonis, root of common peony, angelica sinensis, radix aconiti carmichaeli) on the treatment of heart failure. The compounds of CHMs and corresponding targets were retrieved in TCMSP (http://lsp.nwu.edu.cn/tcmsp.php, *OB* ≥ 30% and *DL* ≥ 0.18), BATMAN (http://bionet.ncpsb.org/batman-tcm/, predicted candidate target proteins with scores ≥20 and adjusted *P* < 0.05), and Pubchem (https://pubchem.ncbi.nlm.nih.gov/). TTD(https://db.idrblab.org/ttd/), OMIM(http://www.omim.org/) and DrugBank(https://www.drugbank.ca/) were used for the retrieval of heart failure related targets. Metascape(http://metascape.org/) was used for metabolic pathway analysis.

Finally, 20 compounds of astragalus with 135 targets, 65 compounds of the root of red-rooted salvia with 218 targets, 22 compounds of safflower with 217 targets, 29 compounds of root of common peony with 190 targets, 7 compounds of ligusticum wallichii with 7 targets, 2 compounds of angelica sinensis with 2 targets, 21 compounds of radix aconiti carmichaeli with 11 targets, 22 compounds of radix ophiopogonis with 243 targets, 22 compounds of ginseng with 76 targets, 12 compounds of semen lepidii with 100 targets, and 150 heart failure related targets were screened out. Then, repeated targets of the CHMs and heart failure related targets were deleted respectively, and 527 targets of the CHMS and 150 targets of heart failure were left. The same targets of CHMs and heart failure were extracted, and 15 targets were obtained (CA2, CACNA1C, CYP2D6, CYP1A2, CYP1A1, AR, CYP2C9, CYP3A4, XDH, ADORA1, ABCB1, CA1, ATP1A1, NR3C2, CACNA1D), indicating that the efficacy of CHM on heart failure maybe related to energy metabolism, regulation of calcium channel, regulation of water electrolyte balance and maintenance of homeostasis.

Furthermore, the 527 targets were imported into Metascape for metabolic pathway analysis, showing that these 10 herbs may affect metabolic process, hormone levels, ion transport, oxidative stress, and homeostatic process, through which CHM may treat heart failure (Table 3). This conclusion is similar to the above one.

**Table 3:** Significant metabolic pathways that are related to these 10 herbs.

**No**.	**Pathway or process**	**Count**	**%**	**Log10(P)**	**Log10(q)**
1	organic hydroxy compound metabolic process	80	15.24	−43.27	−39.27
2	response to inorganic substance	77	14.67	−39.17	−35.35
3	drug metabolic process	92	17.52	−38.76	−35.06
4	cellular response to organic cyclic compound	78	14.86	−36.75	−33.15
5	regulation of hormone levels	73	13.90	−35.79	−32.33
6	cellular response to nitrogen compound	78	14.86	−33.64	−30.30
7	regulation of ion transport	75	14.29	−32.02	−28.76
8	response to oxidative stress	63	12.00	−30.96	−27.74
9	regulation of homeostatic process	65	12.38	−30.25	−27.09
10	regulation of lipid metabolic process	51	9.71	−24.36	−21.61

*“Count” is the number of target genes of the 10 CHMs with membership in the given ontology term. “%” is the percentage of total target genes of the 10 CHMs that are found in the given ontology term. “Log10(P)” is the p-value in log base 10. “Log10(q)” is the multi-test adjusted p-value in log base 10. The metabolic pathways are arranged in ascending order of their respective q-value*.

## Discussion

CHM is regarded by the public and some healthcare providers as effective, gentle and safe (Patil and Kannapan, 2014; Ding and Lian, 2015), and has a good effect on alleviating symptoms and improving well-being of heart failure, including HFpEF (Li et al., 2014). The metabolic pathway analysis of the top 10 used CHMs in this study showed that the efficacy of CHM on heart failure maybe related to energy metabolism, regulation of calcium channel, regulation of water electrolyte balance and maintenance of homeostasis. This review was aimed to evaluate the effect of CHM on quality of life and exercise tolerance in patients with HFpEF. Due to poor study quality and limited data, no definitive conclusions can be drawn from this review on the effectiveness of CHM for HFpEF.

## Summary of main results

The results of this meta-analysis show significant improvements in 6MWD, quality of life, and clinical efficacy rate in HFpEF patients treated with CHM, although there is some heterogeneity between these studies. Compared with conventional western medicines with or without placebo, combined Chinese herbal medicines and conventional western medicines can also reduce all-cause mortality, heart failure hospitalization, and biomarkers (BNP or NT-proBNP), although the results of all-cause mortality were not statistically significant and there is significant heterogeneity between subgroups of BNP. Besides, the completion rate of all studies was above 99% with no severe adverse events, which suggests that CHM might be an effective and safe choice for HFpEF by alleviating symptoms and improving well-being of HFpEF.

## Strengths and limitations

A previous meta-analysis studied the effects of beta-blocker, ACE inhibitors, aldosterone receptor blockers, and mineralocorticoid receptor antagonists on all-cause mortality, cardiovascular mortality, heart failure hospitalization, exercise tolerance, quality of life and biomarkers in HFpEF patients, which showed no effect of any single drug on HFpEF compared with placebo, but the reduction of all-cause mortality and cardiovascular mortality with beta-blocker (Zheng et al., 2018). Another meta-analysis showed that CHM is effective in improving quality of life in patients with chronic heart failure, including HFrEF and HFpEF (Li et al., 2014). However, the efficacy of CHM for HFpEF patients has not been clearly demonstrated. Through quantitative synthesis, our review firstly showed that CHM can improve quality of life and exercise tolerance in patients with HFpEF, which further suggests that CHM could be used in HFpEF treatment as an adjuvant therapy to alleviate symptoms and improve well-being.

However, there are also some limitations in our review. We only performed a search for Chinese and English studies, and it is possible that articles on CHM for HFpEF may have been published in other languages. Moreover, in our review, the composition and dosage of CHM and duration of medication were not considered, which may affect the efficacy. The methodological quality of the included trials was not promising: in addition to the fact that CHM is difficult to be blinded, the included studies have other flaws such as poor randomization and allocation concealment. Since the number of studies in each project is < 10, we did not generate a funnel plot to detect any probable publication bias. According to GRADE system, the evidence of CHM for HFpEF was assessed as very low quality, low quality and moderate quality. Therefore, the evidence in support of CHM in the treatment of patients with HFpEF was inconclusive.

## Implications for research

Although this study shows that CHM may be effective and safe for HFpEF, the current evidence, and potential findings should be interpreted carefully because of poor methodological quality of included studies, insufficient evidence for efficacy and safety, and clinical heterogeneity.

Further research is needed to pay more attention to the efficacy and safety of CHM on HFpEF. Rigorous RCTs with larger sample size and high methodology quality are required to explore the effects of CHM in clinical practice and provide evidence-based data for the promotion of CHM.

## Conclusion

In conclusion, our meta-analysis suggests that compared with conventional western medicine with or without placebo, combined CHM and conventional western medicine might be effective to improve exercise tolerance and quality of life in HFpEF patients, but new well-designed studies with larger sample size, strict randomization, and clear description about detection and reporting processes are needed to further strengthen this evidence.

## Author contributions

JC, WW, and JPW: Conceived and designed the experiments; JPW, RY, FZ, CJ, and PW: Performed the experiments; JL, KG, and HX: Literature searching; JPW, JW, and HZ: Data extraction and risk of bias assessments; JPW, RY, and JC: Analyzed the data; JPW: Wrote the paper: JPW, RY, FZ, CJ, PW, JL, KG, HX, JW, HZ, WW, and JC: Read and approved the manuscript.

### Conflict of interest statement

The authors declare that the research was conducted in the absence of any commercial or financial relationships that could be construed as a potential conflict of interest.
